# Partial Discharge Activity Inductive Sensors and the Application of Magnetic Materials

**DOI:** 10.3390/s25185896

**Published:** 2025-09-20

**Authors:** Ján Zbojovský, Ardian Hyseni, Jaroslav Petráš

**Affiliations:** Department of Electric Power Engineering, Faculty of Electrical Engineering and Informatics, Technical University of Košice, 040 01 Košice-Sever, Slovakia; ardian.hyseni@tuke.sk (A.H.); jaroslav.petras@tuke.sk (J.P.)

**Keywords:** partial discharge, electric power engineering, high voltage, monitoring

## Abstract

The monitoring of partial discharge activity is part of very basic methods used to determine the status of insulation systems in high-voltage electric power devices. These methods use direct galvanic coupled measurement, measurement by inductive offline methods, or other non-electric methods. The inductive method requires sensitive inductive sensors, which detect the partial discharge pulses occurring in high-voltage circuits. The sensitivity of such sensors strongly depends on the design, construction, and materials of the sensor core. Therefore, knowledge of the magnetic material parameters of a sensor is crucial for obtaining optimal values in terms of the sensor’s final sensitivity. In this experiment, experimental sensor cores were constructed using different magnetic materials and different sensor construction processes. For the laboratory experiments, two types of magnetic material were selected: a magnetic material based on Fe-Ni (Permalloy) and a magnetic material based on MO.Fe_2_O_3_. As a reference level for sensitivity, the minimum acceptable sensitivity was defined as the equivalent measuring sensitivity obtained using the direct galvanic measurement method. The optimal construction type and magnetic material for the sensor core of inductive sensors were determined.

## 1. Introduction

A long service life and reliability are fundamental prerequisites for insulating materials and system components used in high-voltage electrical power devices [1]. In usual applications, insulation must withstand constant mechanical stresses, thermal cycling during power generation and transmission, and aggressive chemical environments, as well as moisture ingress [2]. In addition, long-term aging phenomena such as thermal degradation and corona erosion affect the insulation system status [3]. Any deterioration in the insulation system over time can lead to failures, unplanned outages, and costly repairs. Prediction and reliability assurance are important objectives in both material selection and system design [1,4].

High-voltage devices, e.g., electric power transformers, gas-insulated switchgears, and rotating machines, usually have very complex electrode configurations, and their insulation systems have very complex geometric structures. Such structures include non-uniform, ionizing electric fields in regions with sharp edges, voids, or material interfaces [5]. In such non-homogeneous fields, localized field enhancement can produce partial discharge (PD) activity, leading to progressive damage to insulation at microscopic sites [6]. Such activity becomes more intensive in mixed dielectric systems, as solid, liquid, and gaseous insulators are in close proximity, with high field distortion and space-charge accumulation [7].

Partial discharge activity is one of the most sensitive and quantitative indicators of an insulation system’s status and integrity under high-voltage stress [1,8]. PD activity can also cause microscopic defects, e.g., air bubbles, delaminated areas, and sharp corners. In these locations, the local field intensity exceeds the inception threshold for ionization [9]. This leads to further effects, e.g., chemical by-products (ozone and nitrogen oxides), acoustic emissions, and electromagnetic pulses, which also contribute to long-term insulation degradation through chemical attack, carbonization, and erosion of polymeric materials [10]. Monitoring partial discharges over time allows the recording of progress the insulation aging; it also allows the prediction of the remaining life of an electric power device and the scheduling of preventive maintenance [4].

A wide range of PD activity detection and monitoring techniques have been developed. Electrical methods detect high-frequency currents induced at a piece of equipment’s terminals [11], acoustic sensors detect ultrasonic emissions [12], optical systems capture light from coronae [13], and chemical sensors measure byproduct concentrations in transformer oil [14]. Among these, inductive (or high-frequency current transformer) sensors are widely used for online monitoring, thanks to their non-invasive installation and compatibility with live equipment. The sensors encircle a ground or phase wire conductor and detect the current pulses generated by PD activity [15].

The sensitivity and fidelity of inductive PD sensors hinge critically on both their mechanical construction and the magnetic properties of the core material [16]. Core permeability, saturation flux density, loss characteristics, and temperature stability all influence a sensor’s ability to detect weak, high-frequency PD pulses against a background of electromagnetic interference [17]. Materials such as nanocrystalline alloys, amorphous metal ribbons, and specialized ferrites have been employed to optimize performance; nanocrystalline cores offer very high permeability and low core losses, while certain ferrites provide excellent attenuation of unwanted low-frequency noise. The choice of a particular magnetic alloy or composite must balance sensitivity requirements with factors like thermal management, mechanical robustness, cost, and compatibility with the sensor housing [18,19].

Currently, especially in the past five years, significant progress has been made in the research of magnetic materials used in the sensors used for partial discharge monitoring and detection using the inductive method. Qian et al. [20] demonstrated that the addition of BaTiO_3_ to NiCuZn ferrites leads to an increase in permeability and sensitivity at high-frequency pulses, which is advantageous for the construction of highly sensitive current transformers. In another article, Li et al. [21] proposed a magnetoelectric sensor using piezoceramics (PZT) in combination with an amorphous alloy Metglas, which allows the detection of high-frequency current pulses with very low noise. Fritsch and Wolter [22,23] studied the saturation properties of sensor cores and proposed an optimized design of a split core with a controlled air gap. Further research was focused on sensor geometries with flexible nanocrystalline alloy strips [24] and loop antennas without resonance effects [25], as well as sensors with optimized core shapes for installation in real conditions. These approaches show new possibilities for increasing the sensitivity, frequency range, and reliability of PD signal sensing while maintaining compatibility with existing power equipment.

In addition to material selection, sensor design considerations include winding geometry, shielding against external electromagnetic interference, impedance matching to signal processing circuitry, and the use of broadband amplifiers to reproduce the fast PD pulses. Advances in additive manufacturing and thin-film deposition techniques now allow the creation of core geometries and multi-layered magnetic composites, further enhancing sensor responsiveness. Ultimately, achieving optimal sensor sensitivity involves a holistic approach that integrates core material science, precision engineering, and signal processing, ensuring that the discharge activity is reliably detected and quantified.

In the experiments, experimental sensor cores were constructed using various magnetic materials and different sensor designs. For the laboratory investigations, two types of magnetic materials were selected: one based on Fe-Ni (Permalloy) and the other based on MO.Fe_2_O_3_. As a reference level for sensitivity, the minimum acceptable sensitivity was set as the equivalent measuring sensitivity obtained using the direct galvanic measurement method. The optimal sensor core construction and the most suitable magnetic material for inductive sensors were determined.

## 2. Inductive Sensor Material and Construction

For an in-depth analysis of the material used for the sensor and the analysis of the construction, it is necessary to look at Equation (1), which defines the reciprocal coefficient of the induction and can be calculated as follows:

(1) $$M_{12}=\frac{\mu_{0}\mu_{r}}{2\pi }N_{2}h\ln\frac{r_{2}}{r_{1}}$$

where

*μ*_0_ is the permeability of free space;

*μ*_0_ = 4*π* × 10^−7^ H/m;

*μ*_r_ is the relative permeability of the material between the coils (≈1 for air or vacuum; much larger for ferromagnetic cores);

*N*_2_ is the number of windings on the secondary coil, whose flux linkage we are calculating;

r_2_—outer radius of the core;

r_1_—inner radius of the core;

*h* is the axial length of both coils, assumed long enough that end effects can be neglected and the field is uniform along h.

ln(r_2_/r_1_) arises because the magnetic field between coaxial cylinders falls off.

In short, the derivation of Equation (1) is as follows.

The magnetic field between the cylinders, carrying the current I_1_, is calculated as in Equation (2):

(2) $$B\left(r\right)=\frac{\mu_{0}\mu_{r}I_{1}}{2\pi \;r},\;\;r_{1}<r<r_{2}$$

where

r_2_—outer radius of the core;

r_1_—inner radius of the core;

r—variable radius of the core;

*μ*_0_ is the permeability of free space;

*μ*_0_ = 4*π* × 10^−7^ H/m;

*μ*_r_ is the relative permeability of the material between the coils;

I_1_—the current flowing though the wire;

B—magnetic flux density.

The magnetic flux through one turn of the secondary coil over height h can be calculated from Equation (3):

(3) $$\Phi =\int_{r1}^{r2}B\left(r\right)\;h\;dr=\;\frac{\mu_{0}\mu_{r}I_{1}\;h}{2\pi \;}\;\int_{r1}^{r2}\frac{dr}{r}=\;\frac{\mu_{0}\mu_{r}I_{1}\;h}{2\pi \;r}\;\ln\frac{r2}{r1}$$

where

Φ—magnetic flux;

B—magnetic flux density;

r_2_—outer radius of the core;

r_1_—inner radius of the core;

r—variable radius of the core;

h is the axial length of both coils.

Then the mutual inductance M_12_ is calculated as flux per turn times number of turns, divided by I_1_, in Equation (4):

(4) $$M_{12}=\frac{N_{2}\;\Phi }{I_{1}}=\frac{{µ}_{0}{\;µ}_{r}}{2_{\pi }}{\;N}_{2}h\;\ln\frac{r_{2}}{r_{1}}$$

where

M_12_—mutual inductance;

N_2_—number of loops in the secondary coil;

Φ—magnetic flux;

*μ*_0_ is the permeability of free space;

*μ*_0_ = 4*π* × 10^−7^ H/m;

*μ*_r_ is the relative permeability of the material between the coils;

h is the axial length of both coils;

*r*_2_—outer radius of the core;

*r*_1_—inner radius of the core;

I_1_—the current flowing though the wire.

Therefore, higher *μ*_r_ means better magnetic core parameter and boosts M_12_. The more windings N_2_, the higher the flux. In addition, a longer overlap h increases the total flux. The logarithmic dependence on the radii ratio means that widening the gap between coils weakens coupling logarithmically.

The expression for the self-inductance is given by Equation (5):

(5) $$L_{2}=\frac{\mu_{0}\mu_{r}}{2\pi }N_{2}^{2}h\ln\frac{r_{2}}{r_{1}}$$

where

L_2_—the self-inductance;

N_2_—number of loops in the secondary coil;

*μ*_0_ is the permeability of free space;

*μ*_0_ = 4*π* × 10^−7^ H/m;

*μ*_r_ is the relative permeability of the material between the coils;

*h* is the axial length of both coils;

r_2_—outer radius of the core;

r_1_—inner radius of the core.

The self-inductance L_2_ of a long, coaxial cylindrical winding with N_2_ turns, height h, inner radius r_1_, outer radius r_2_, and core permeability *μ*_0_*μ*_r_, is determined by this equation.

Based on the geometry, a single coil of length h is considered. The windings are assumed to form a cylindrical layer between r_1_ and r_2_. For a long solenoid or cylindrical winding, the magnetic field inside the winding is assumed to be equivalent to that derived from Equation (2).

In the coil with N_2_ turns, the same magnetic flux is assumed to pass through each turn (under the assumption of tight packing and identical enclosed area). The total flux is then calculated as described in Equation (3).

However, the definition of self-inductance is determined by Equation (6):

(6) $$L_{2}=\;\frac{N_{2}\Phi }{I}\;\frac{\mu_{0}\mu_{r}}{2\pi \;}{\;N}_{2}h\;\ln\frac{r_{2}}{r_{1}}\;\times N_{2}=\frac{\mu_{0}\mu_{r}}{2\pi \;}{\;N}_{2}^{2}h\;\ln\frac{r_{2}}{r_{1}}$$

The factor of N_2_, in comparison to the reciprocal-inductance case, is introduced to account for the fact that each turn contributes to and links with the total magnetic flux generated by all turns.

*μ*_0_ is the permeability of free space (4π × 10^−7^ H/m).

*μ*_r_ is the relative permeability of the core material (≈1 for air; ≫1 for ferromagnetic cores).

N_2_^2^ is the squared winding count—self-inductance scales with the number of loops squared because both the flux generated and the number of turns linking that flux grows with this parameter.

h is the axial length; more length means more linked flux.

ln(r_2_/r_1_) is the geometric factor from integrating the field across the radial construction of the winding; wider radial thickness means more stored energy logarithmically.

Therefore, an increase in the winding number N_2_ results in a quadratic increase in the inductance L_2_. Longer coils are associated with greater magnetic energy storage per unit current, thereby increasing L_2_. Tighter radial packing (i.e., a smaller ratio r_2_/r_1_) leads to a slight reduction in inductance; however, due to the logarithmic dependence, further reduction in coil thickness yields diminishing returns. The use of high-permeability core materials leads to flux concentration and can increase L_2_ by several orders of magnitude when the relative permeability *μ*_r_ is sufficiently high. This compact formula is commonly applied in the design of coaxial inductors, toroidal shells, or tightly wound cylindrical coils, where end effects are negligible and the windings form a uniform annular structure. The transformation coefficient is defined as *k* = M_12_/L_2_ = 1/N_2_ and the following parameters are used in the calculation:

*µ*_0_ = 1.253 × 10^−6^ H/m;

*µ*_r_—relative permeability of the toroidal core material;

N_2_—number of secondary coil windings;

*H*—height of the toroidal structure;

*r*_1_, *r*_2_—inner and outer radius of the toroid.

The special feature of this construction of the sensor is the independence of the transformation coefficient on the form of the primary wire. The transmission function can be calculated using Equation (7):

(7) $$\frac{u_{2}}{i_{1}}=\frac{M_{12}R}{L_{2}}.\frac{1}{\sqrt{1+{\left(\frac{R}{{\omega L}_{2}}\right)}^{2}}}=\frac{R}{N_{2}}.\frac{1}{\sqrt{1+{\left(\frac{R}{{\omega L}_{2}}\right)}^{2}}}$$

where

u_2_—voltage on the secondary coil;

i_1_—the current flowing through the primary coil (in the high-voltage circuit);

L_2_—self-inductance of the secondary coil;

M_12_—mutual inductance;

R—terminating impedance of the secondary coil;

ω—angular frequency;

N_2_—number of secondary coil windings.

As can be observed, the transmission function exhibits a frequency dependence, which implies that the inductance of the circuit varies according to the permeability of the core material. Moreover, the relative permeability *μ*_r_ itself is also frequency-dependent. Based on the previously introduced equations, it can be stated that operating conditions are fulfilled for small alternating or pulsed currents. The input of the secondary winding behaves as an electrical circuit with high input impedance. A reduced number of turns ensures greater immunity to external noise signals.

For alternating current operation, non-retentive core materials with a coercive force *H*_c_ > 800 A/m are considered suitable. These materials are characterized by narrow hysteresis loop and exhibit both high initial permeability *μ*_p_ and high maximum permeability *μ*_m_. Furthermore, the advantage of the designed inductive sensor is that it can also measure the leakage current of the insulation system at the frequency of 50 Hz, which is another parameter indicating the status of the insulation material.

During the measurement of partial discharge signals using inductive sensors, it is assumed that the core material remains unsaturated, i.e., it operates under very low magnetic field strength *H*. The initial permeability of the core has a substantial impact on the measurement sensitivity and is dependent on both temperature and frequency. Experimental results have shown that the relative permeability *μ*_r_ of the tested materials changes significantly at the frequencies above 5 × 10^5^ Hz.

**Figure 1:**
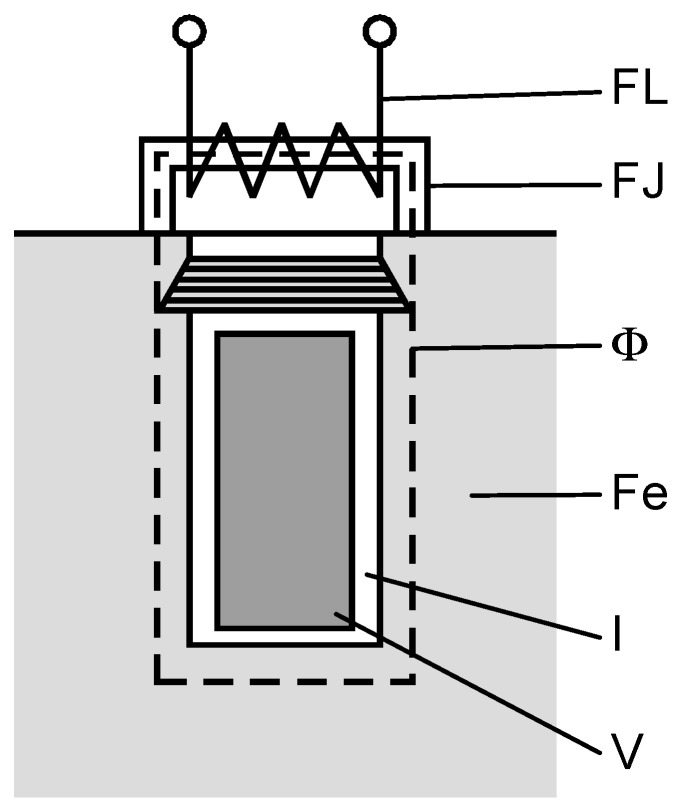
Inductive sensor—the drawing.

**Figure 2:**
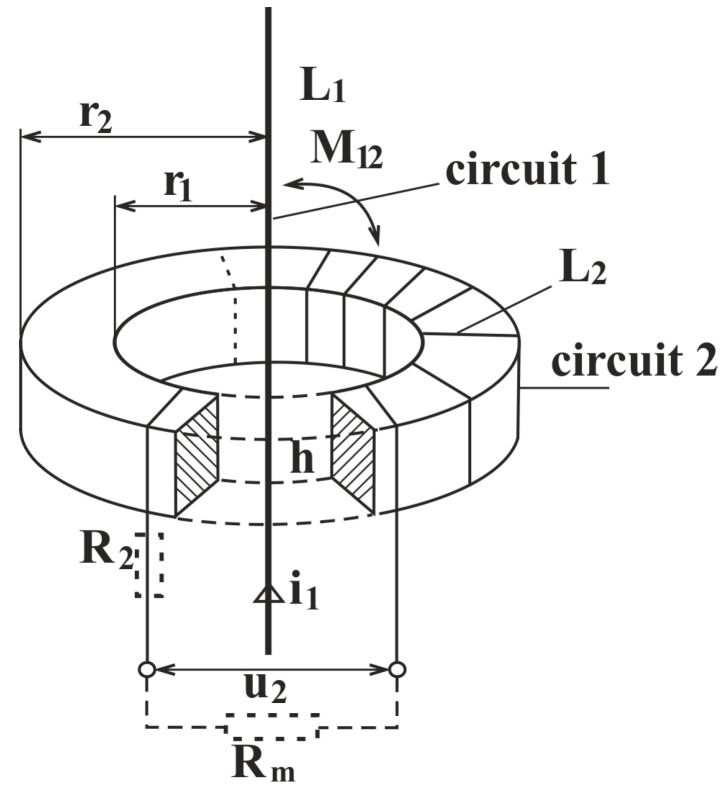
Inductive sensor—the electro-magnetic situation.

A sensor design has been proposed based on the consideration that it operates as part of the electrical circuit of the device under test. The layout and construction of the sensor are depicted in Figure 1 and Figure 2. A model of the winding is presented in the subsequent two figures. According to the configuration of the generator pole within the stator drainage area, and after certain technological adjustments, the placement of the inductively coupled sensor is illustrated in Figure 1.

In Figure 1, the parts of the sensor are as follows:

FL—sensor winding;

FJ—sensor;

Φ—magnetic flow;

Fe—stator steel plate;

I—insulation;

V—Cu wire.

Figure 2 shows the electromagnetic situation in the toroidal inductive sensor in our experiments. The primary conductor with the current i_1_ passes through the center of the toroid. This current produces a magnetic field and is guided by the magnetic core between the inner radius r_1_ and the outer radius r_2_, all along the axial height h. A secondary winding is placed on the core with the self-inductance L_2_. The mutual inductance with the primary conductor is denoted as M. The induced voltage on the secondary coil is denoted as u_2_. The winding has a resistance R_2_ and the measuring circuit includes an external measuring resistance *R_m_*. The figure demonstrates the magnetic coupling path between two electric circuits.

In summary, in Figure 2, the parts of the sensor are as follows:

r_2_—outer radius;

r_1_—inner radius;

L_1_—self-inductance of the wire;

M_12_—reciprocal inductance;

L_2_—self-inductance of the windings;

h—height of the core;

i_1_—current flowing through the wire;

u_2_—voltage generated by the sensor;

R_2_—resistance of the winding;

R_m_—measuring resistance.

For our laboratory case, we have chosen the sensor construction with the following dimensions of the core: r_1_ = 40 mm; r_2_ = 60 mm; h = 14 mm. The winding was one-layered with CuL wire with a diameter of 0.18 mm and with 66 turns in the layer. The core was first insulated by a layer of varnish. Then, the winding was made and after 10 loops, the varnish was applied for fixing the loops.

**Figure 3:**
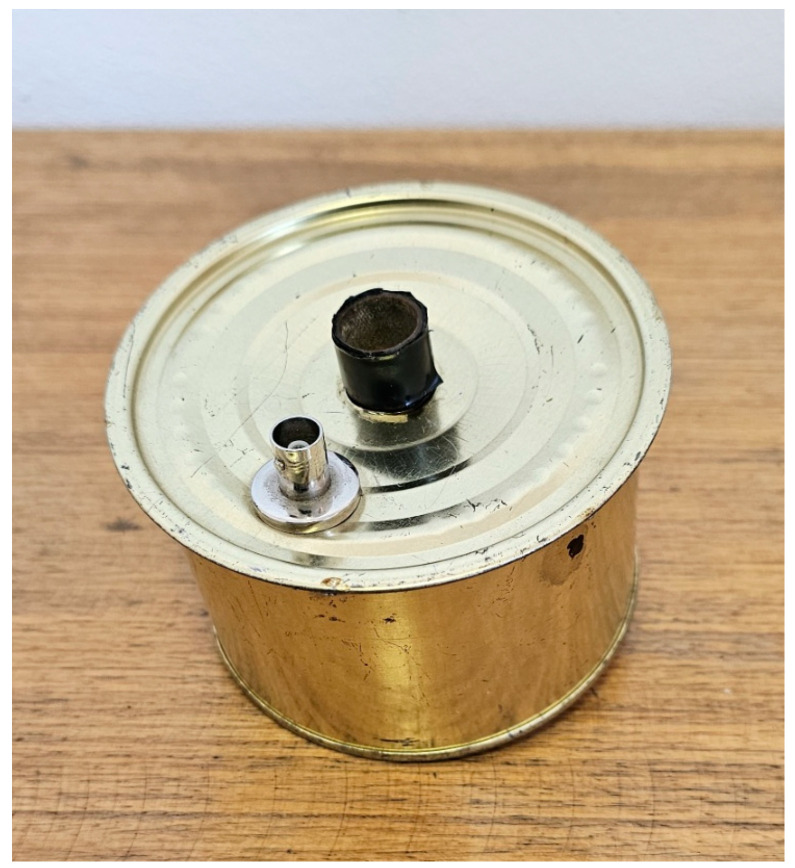
Inductive sensor encapsulated in the protective case.

Figure 3 shows our assembled inductive sensor encapsulated in a protective case. This sensor sample was assembled only for laboratory purposes.

## 3. The Experimental Setup

**Figure 4:**
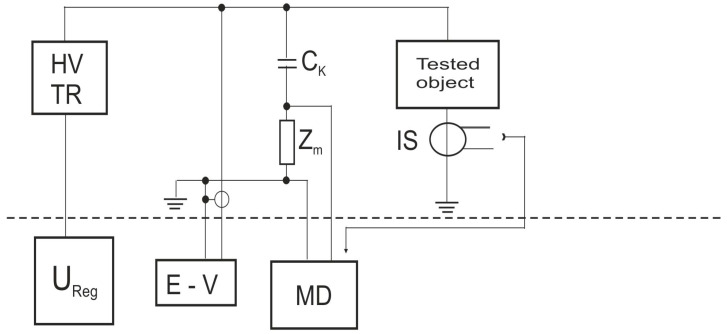
The experimental setup of the inductive sensor measurement method.

The experimental setup can be seen in Figure 4.

The electrical block diagram parts are as listed:

HV—high-voltage transformer;

U_reg_—voltage regulator;

E-V—electro-static voltmeter;

IS—inductive sensor;

C_k_—coupling capacity;

MD—partial discharge measurement device.

A series of experiments was conducted for each selected sensor core material. The tested object was a high-voltage coil from a stator winding. All measurements were performed under laboratory conditions in accordance with the IEC 60270 standard [26], with the tested object properly earthed. IEC 60270 defines high-voltage test techniques for partial discharge (PD) measurements, specifically addressing localized discharges occurring in insulating materials, which only partially bridge the dielectric between conductors.

**Figure 5:**
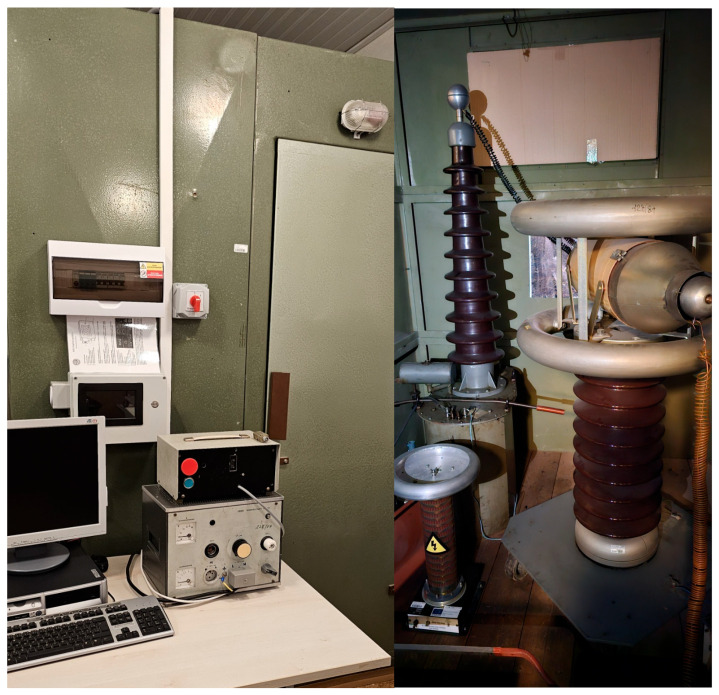
Our experimental setup—low voltage part on the left and high-voltage part on the right side.

In Figure 5 our laboratory experimental setup is depicted, with the low-voltage part (regulator and measurement device) on the left side and the high-voltage part (transformer, coupling capacitor) on the right side.

Simultaneously with the inductive method, direct galvanic measurements were performed for comparison and validation purposes. This method is recognized as reliable and is widely used in industrial practice for evaluating basic parameters of partial discharge phenomena.

During the experimental procedure, the test coil was placed on an insulating support to prevent contact with surrounding objects and to eliminate parasitic partial discharges. In order to suppress corona discharges, a homogenization electrode was installed at the high-voltage end of the winding. The grounded end of the coil served as the drain terminal.

The number of experiments for both the galvanic and inductive method series were approximately 50 for each method.

In each experiment in the series, the tested voltage was set to the initial value and then increased until the initiation voltage level, i.e., the voltage at which the first partial discharges occurred. At this voltage level, partial discharge parameters were stored by the measurement device with dedicated and customized software. Then, the voltage was increased by 500 V step until the nominal voltage level of 5 kV. At each voltage level, partial discharge parameters were measured and stored repeatedly. This approach ensures that the sensors are tested in a wide voltage range because at each voltage, the partial discharge activity is different for the same tested voltage. It was important to ensure that the voltage only rises and does not fluctuate during one measurement.

In the inductive measurement phase, the tested sensor was placed in a magnetically shielded enclosure and connected within the operational earthing path of the high-voltage system. The voltage increase procedure mirrored that of the galvanic method, and at each voltage level, PD activity was recorded for subsequent analysis.

Two magnetic materials were preselected for use in the sensor core; the first one was a magnetic material based on Fe-Ni (Permalloy) and the second one was a magnetic material based on MO.Fe_2_O_3_.

When comparing these materials, attention was focused on their permeability as a function of frequency.

The Permalloy (Fe–Ni) has a very high initial permeability (*μ*_1_ = 5000–10,000), which is advantageous for low frequencies below 100 kHz, but its permeability drops off quickly above MHz frequencies. The material for the sensor based on MO.Fe_2_O_3_ Ferrite has a moderate permeability *μ*_1_ = 200–1000 and it remains relatively flat up to tens or hundreds of MHz. For this material, the core losses at frequencies usual for partial discharge pulses are small.

In terms of electrical resistivity, Permalloy is a low-resistivity material (~10^−7^Ωm), which allows the formation of strong Foucault currents under high-frequency excitation. This effect damps the signal and broadens the impulse response, resulting in reduced resolution for sub-nanosecond partial discharge pulses.

The MO.Fe_2_O_3_ Ferrite material is a ceramic metal-oxide with high resistivity 10^2^–10^4^ Ω·m. Also, the Foucault’s current at PD frequencies should not occur, so the coil transforms the discharge pulse with minimal or no distortion.

For the decision of material selection, we also performed preliminary core material experimental checks of the hysteresis loop and Foucault currents loss. For these experiments we used a laboratory setup without specialized hysteresis or impedance analyzers; therefore, we could only obtain their approximate characters, but for our proposal, the approximation was enough to make a decision. For the hysteresis loop and the parameters of it we used a two-winding method with a toroidal construction of excitation coil and a measurement coil and with a power source, an oscilloscope, and an integrator. The sample core was wound with a primary winding (N_1_ = 100 loops) and a secondary winding (N_2_ = 100 loops) and the measurements were made at room temperature (25 °C). The value of saturation magnetic flux B_sat_ = 0.65 T for Permalloy core material and B_sat_ = 0.35 T for our sample of core material MO.Fe_2_O_3_. The values of coercivity H_c_ = 1.6 Am^−1^ for Permalloy and H_c_ = 10 Am^−1^ for MO.Fe_2_O_3_. Furthermore, the values for initial permittivity μ_i_ = 90,000 for Permalloy and μ_i_ = 2000 for MO.Fe_2_O_3_.

For current loss approximate measurements, we used a method with a sinus signal generator with changeable frequency, a precise referential resistor, and 2-channel oscilloscope. The loss factor tanδ includes the eddy current loss and hysteresis loss. For the Permalloy core material, the loss factor at 100 kHz was 0.018 and at 1 MHz was 0.185, so there was an increasing trend for higher frequencies. In the case of the MO.Fe_2_O_3_ core material, we had the loss factor at 100 kHz equaling 0.013 and at 1 MHz it was 0.042, with a less steep increasing trend at higher frequencies.

**Figure 6:**
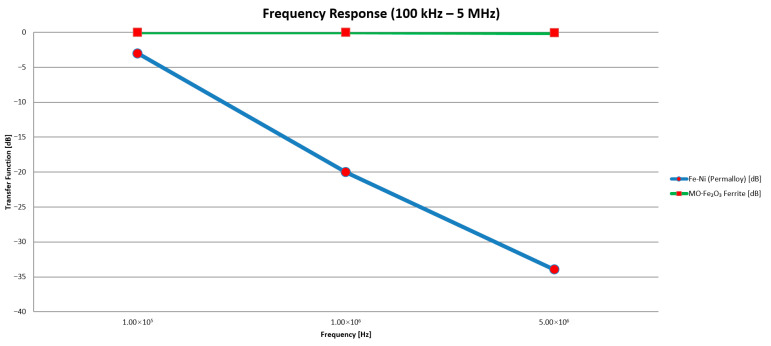
The frequency response of both magnetic core materials.

In Figure 6, the frequency response for both materials can be seen. This was the key point in the preliminary decision regarding which core material to use for encapsulated inductive sensor construction. It is obvious that for the Permalloy core material, the frequency response curve decreases with the increasing frequency. This indicates high signal attenuation in the frequency range of the partial discharge pulse spectrum causing possible signal wave-shape distortions. This is crucial for signal evaluation at later stages.

In summary, a trade-off had to be made between sensitivity and bandwidth. While the Permalloy core offered higher permeability, it also introduced significant signal distortion due to limited bandwidth. The ferrite material, on the other hand, provided lower permeability but offered a broader and flatter frequency response, along with lower core losses—attributes beneficial for accurate pulse shape reproduction in PD diagnostics.

Therefore, the MO.Fe_2_O_3_ ferrite was selected as the core material for the laboratory sensor. The partial discharges typically contain high-frequency components and small charge magnitudes. The ferrite core offered sufficient permeability to enable signal detection and amplification, combined with high resistivity to suppress the Foucault’s currents, and a wide, flat frequency response to preserve the amplitude and timing of PD events.

The tested inductive sensors were connected as shown in Figure 4. The sensor is denoted as IS (inductive sensor) and it is connected to the input of the preamplifier of the measurement device (MD). The coupling capacitance and measuring impedance, together with the measurement object, create the basic high-voltage circuit. The high-voltage transformer (HV TR) supplies the circuit with high voltage, which is measured by an electrostatic voltmeter (EV). The high voltage level is adjusted by a regulator.

## 4. Results

Based on the stored measurement data and the results of statistical evaluation, including phase-resolved analysis using appropriate statistical methods applied to each dataset, a series of graphs representing the partial discharge (PD) characteristics were obtained:

1. The partial discharge phase distribution;
2. The sum of charge per half wave of the testing voltage;
3. Apparent charge mean value phase distribution;
4. Apparent charge amplitude spectrum;
5. Apparent charge maximal value phase distribution.

A customized measuring device, equipped with dedicated software for the statistical evaluation of partial discharge (PD) pulse signals, was employed to obtain all of the aforementioned PD activity parameters. In addition, the distributions of the maximum and the mean values of the apparent charge, discharge rate, and amplitude spectrum were calculated. All the charge and apparent charge units were stated in pC (picocoulomb, 1 pC = 10^−12^ coulomb).

For the comparison of performance, we used the parameters charge and apparent charge. Obviously, there is a difference between these parameters; the measured charge is the actual charge measured by the measuring circuit during a partial discharge occurrence. This is influenced by the measuring circuit itself, the impedance of the measurement device input, the value of the measurement resistor, and the connection setup. This makes the value instrument-dependent. The apparent charge, as defined by IEC 60270, is the equivalent charge that, if injected directly at the test object’s terminals, would produce the same voltage change as the discharge, providing a standardized and system-independent measure of PD magnitude.

**Figure 7:**
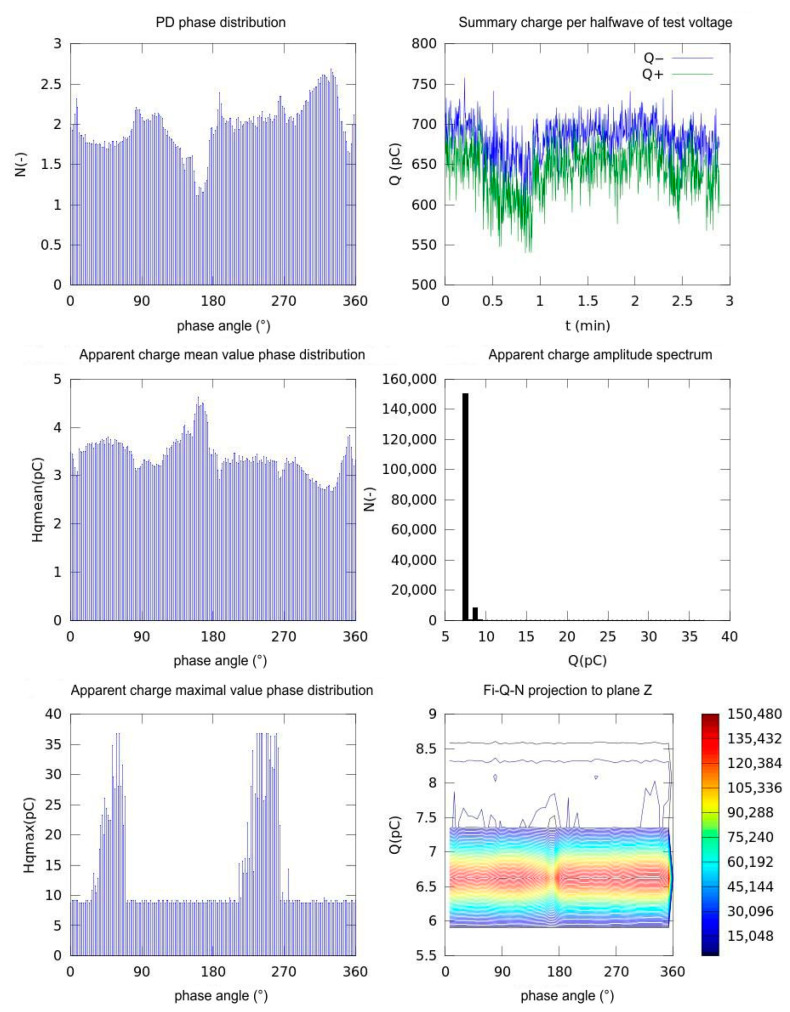
Partial discharge activity parameters at nominal testing voltage 2.5 kV, galvanic method.

**Figure 8:**
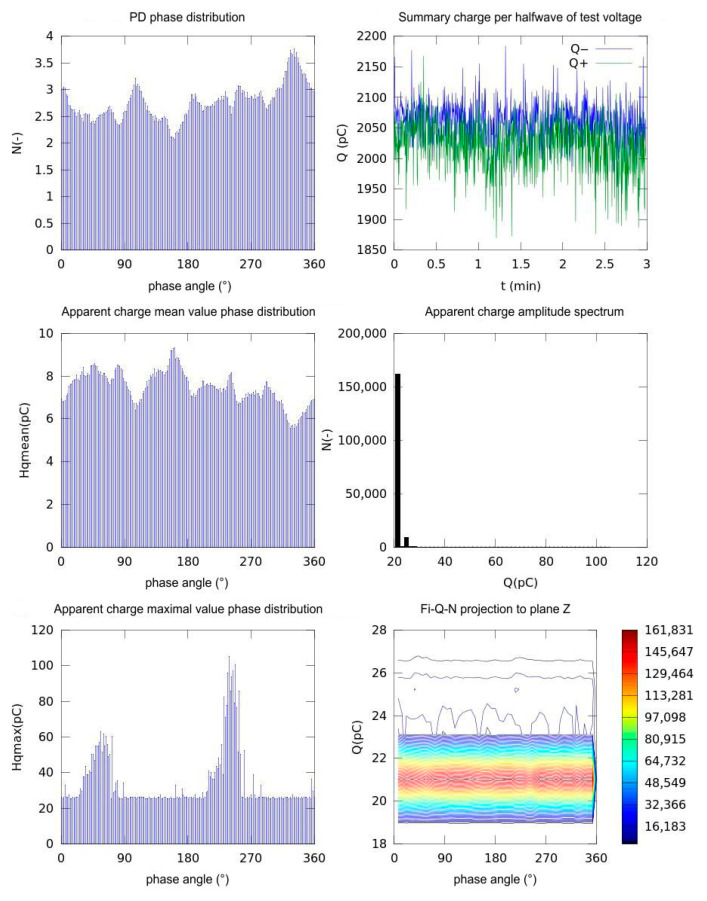
Phase distribution of partial discharge at nominal voltage 2.5 kV, inductive method, MO.Fe_2_O_3_ sensor material.

In Figure 7, the reference set of graphs obtained from PD activity measurements using the direct galvanic method is presented. In Figure 8, the corresponding graphs obtained using the inductive method with the first selected core material, MO.Fe_2_O_3_, are depicted.

The PD phase distribution shows the number of discharge pulses as a function of the AC testing voltage angle (0–360°). Certain peaks can be found at approximately ~60–100° (in positive polarity) and ~240–280° (in negative polarity). These are the phases in which the voltage reaches its amplitude values and the dielectric material is most stressed electrically. The summary charge per half-wave shows the total accumulated charge (Q^−^ for negative half-waves, Q^+^ for positive half-waves) in each half-cycle over about 3 min. The blue curve (Q^−^) hovers around 700 pC, the green curve (Q^+^) around 650 pC, with only slight fluctuations. We cannot find any clear time trend (no progressive degradation) and the discharge activity remains relatively stable over time. Apparent charge mean value phase distribution shows the average charge per individual pulse (H mean) for each 5° phase interval. The highest mean (~4.5 pC) occurs around ~160–180°, the lowest (~2.8 pC) around ~260–280°. This again matches the two polarities, showing not only when pulses occur most often but also when they are, on average, strongest.

The apparent charge amplitude spectrum is displayed as a histogram of all individual pulse charges Q. The majority of discharges are between 5 and 10 pC; those pulses above 15 pC are relatively rare, and there are none above 20 pC. This is typical for small partial discharges. The apparent charge maximal value phase distribution has the maximum charge in each phase range (H_max_), shown as a histogram. There are sharp spikes around ~80° and ~260°, where occasional stronger pulses reach up to ~35–40 pC. Between those peaks, H_max_ stays consistently low (~8–10 pC).

ϕ-Q-N projection onto the Z-plane is displayed as a 2D density plot showing how the acquired pulses distribute across phase/charge/count space. The colors indicate frequency (red = high, blue = low). A clear band around Q ≈ 6.5 pC shows that most pulses occur at this charge regardless of phase, with local bulges at the noted phases around 90° and 270°.

In summary, these graphs confirm that partial discharges are strongly synchronized with the AC voltage, most frequent and energetic near the voltage peaks and polarity reversals (~90° and ~270°). However, the bulk of discharges is small (5–10 pC), while stronger events appear only occasionally at those critical phases. This phase-resolved PD analysis helps separate steady low-level activity from rare, higher-energy pulses that could signal progressive insulation degradation.

For the inductive measurement method employing the core material MO.Fe_2_O_3_, a comparison of the obtained statistical results is provided in the graphs shown in Figure 8.

The phase pulse count in comparison to the galvanic method shows that the peaks achieve approximately 3.3–3.7 pulses per bin at the same phase angles. The inductive method appears to be more sensitive to the smallest discharges. The total charge per half-cycle in this case has the value peaks at 2050 pC level for the negative half cycle and 2000 pC for the positive half cycle. The time stability is roughly the same as for the galvanic method, but the absolute charge is approximately three times higher due to the sensor calibration factor.

The mean pulse charge according to the testing voltage phase has a similar waveform shape; however, in this scale, it is scaled up to approximately 5.5–9 pC. The pulse partial discharge signal amplitude spectrum is shifted to 20–25 pC, with small amplitude for charges above 50 pC. The histogram shape mirrors that of the shift to higher charge values.

The maximum pulse charge according to the testing voltage phase has higher peaks up to 60 pC in the first polarity and up to 110–12 pC in the second polarity but with identical phase windows.

ϕ-Q-N (Fi-Q-N in Figure 8) projection onto the Z-plane has the same band but it is centered at 21 pC. The contour topology is identical. Both methods agree perfectly on when discharges occur and how their relative magnitudes vary with phase. The inductive probe simply reports every discharge as a larger apparent charge (and catches more of the tiniest events), owing to its coil geometry and electronics. In practical applications, either a calibration factor may be applied to align the absolute values of *Q*, or the focus can be placed on analyzing the shape and phase correlation of the distributions rather than their raw amplitude values.

The summary and overview of the measurement statistical parameters can be seen in Table 1.

In Table 2, we summarize the parameters for comparison, which quantify the ferrite sensor sensitivity and suitability in comparison with the direct galvanic method.

Reading from the graphs and statistical values, the minimum detectable charge can be read from the lowest non-zero bins of the apparent-charge distributions. For the galvanic method, individual PD pulses can be seen at approximately 3 pC, whereas the inductive ferrite sensor shows higher absolute values at 5–6 pC, but it captures 30–40% more pulses at testing voltage phase peaks (3.3–3.7 vs. 2.5–3.0 pulses/bin), indicating higher event-level sensitivity. The peak-to-mean ratio of 8–9 for the galvanic method and 7–13 for the inductive method shows the dynamic range comparison.

## 5. Discussion

Upon analysis of both series of graphs and comparison of the corresponding statistical parameters and observed trends, it can be concluded that both measurement methods are comparable in their ability to detect partial discharge activity. The inductive sensor shows slightly larger apparent charge values; thus, the sensor is more sensitive.

The phase-dependent measurements obtained by both galvanic and inductive methods show identical phase patterns, e.g., peaks in pulse count and charge at 90° and 270° and near 180°, which shows that both techniques record partial discharge activity pulses with comparable sensitivity and precision. However, the inductive sensor shows apparent charge values approximately three times higher than the galvanic sensor in every metric in the graph series. This scaling comes from the inductive sensor calibration constant

The MO.Fe_2_O_3_ ferrite core for the sensor (μ_1_ ≈ 200–1000) gave a broad and flat permeability level up to tens or hundreds of MHz, while its high resistivity (10^2^–10^4^ Ω·m) suppresses Foucault’s currents. This combination yields minimal pulse-shape distortion, a wide detection bandwidth, and sufficient amplification of the tiny PD signals.

The trade-off between permeability and bandwidth is crucial for accurate charge-amplitude and timing measurements. Our experiments confirmed that the MO.Fe_2_O_3_-cored inductive sensor meets or exceeds the minimum sensitivity benchmark set by the galvanic reference (measured according to IEC 60270) across the 500 V–5 kV range, while faithfully reproducing the true shape and timing of PD pulses.

The three-times-higher factor reflects the sensor transfer function and calibration rather than a change in the true charge values. However, using identical conditions, equal metrics show a tripled scaling. The inductive sensor also acquires approximately 30–40% more events at testing voltage phase peaks. This means higher event-level sensitivity for small PD pulses.

It should be noted that the presented tests were conducted in a controlled laboratory environment using stator winding samples under stable humidity and temperature conditions. In contrast, real-world industrial environments typically involve varying temperatures, mechanical vibrations, and electromagnetic interference, all of which may influence sensor performance.

Therefore, further work is proposed to validate the performance of the MO.Fe_2_O_3_-based inductive sensor design under in situ conditions on live high-voltage equipment. Future investigations should also address temperature compensation strategies and the development of robust mechanical packaging to ensure long-term operational reliability.

Our research was intentionally focused on laboratory experiments with laboratory conditions to provide a controlled comparison of different magnetic core materials and winding configurations. This setting allowed us to establish a reliable sensitivity benchmark against the galvanic reference method, in line with IEC 60270, and to eliminate external factors such as temperature fluctuations, vibrations, and electromagnetic interference. Establishing this baseline was a necessary first step before moving to more complex operational environments.

However, the intended usage of the device in industry is the on-line monitoring of high-voltage power equipment, where partial discharge diagnostics are critical for predictive maintenance. Typically, the applications include the following:

- Rotating machines (generators, motors), where the sensor can be clamped around stator winding terminals or grounding conductors without interrupting operation;
- Power transformers, where inductive sensors can be mounted on bushing tap leads or grounding connections to provide continuous PD surveillance;
- Gas-insulated switchgear (GIS) and cable terminations, where space constraints and the need for non-invasive techniques make compact inductive sensors advantageous.

The advantage of the inductive method is that it requires no galvanic connection with the high-voltage circuit; therefore, its electrical safety and simple installation are highlighted.

## 6. Conclusions

Measurements by direct galvanic and indirect inductive methods on coils of electric motors were made for comparative analysis. It was observed that the indirect measurement method using sensors with materials such as magnetic material based on Fe-Ni (Permalloy) and magnetic material based on MO.Fe_2_O_3_ provides sufficient sensitivity needed for partial discharge signal acquisition with all important information in the signal retained for statistical evaluation of partial discharge activity.

The statistical analysis of the recorded PD signals confirmed that both measurement approaches are comparable, provided that a suitable core material is used in the inductive sensor. Both galvanic and inductive methods yield identical PD phase-angle distributions. The sensitivity appears to be higher for the inductive method.

The selected material (MO.Fe_2_O_3_) for inductive PD sensing offers a flat high-frequency permeability response and adequate permeability to amplify sub-nanosecond pulses without distortion.

When looking at the state-of-the-art technologies in the field of partial discharge measurements methods and the electric device diagnostics methods, our research experiments and conclusions contribute to the development of new possible materials and designs for these sensors. Our work compares different magnetic core materials (Fe–Ni Permalloy and MO.Fe_2_O_3_) with specific sensor constructions to identify their impact on the sensitivity of inductive sensors for partial discharge monitoring. Using direct galvanic measurement as a reference benchmark, our study shows that making the right material and sensor design choices, and thus optimizing the inductive sensor parameters, can influence the measurement method performance and achieve comparable sensitivity for a non-intrusive on-line, safe monitoring approach in high-voltage insulation systems. By focusing on the optimal core material and construction, our work also advances the technical field by providing design guidelines for next-generation inductive PD sensors that offer improved sensitivity, reliability, and applicability in industrial diagnostics.

## Figures and Tables

**Table 1:** Sensor performance overview—direct galvanic and indirect inductive method comparison.

Parameter	Galvanic Method	Inductive Method
Q^−^ (negative half-wave)	~700 pC	~2050 pC
Q^+^ (positive half-wave)	~650 pC	~2000 pC
Pulse density (N)	Max. ~2.5–3 pulses/bin	Max. ~3.3–3.7 pulses/bin
PD phase distribution	Peaks at ~60–100° and ~240–280°	Peaks at ~90–100° and ~300–330°
Apparent charge mean value (H_mean_)	~2.8–4.5 pC	~5.5–9 pC
Apparent charge amplitude spectrum	5–10 pC dominant, none >20 pC	20–25 pC dominant, some >50 pC
Apparent charge maximal value (H_max_)	Up to 35–40 pC	Up to 60 pC/110–120 pC
ϕ-Q-N projection	Band at ~6.5 pC	Band at ~21 pC

**Table 2:** Summary of sensitivity parameter comparison: direct galvanic and indirect inductive method comparison.

Parameter	Galvanic Method	Inductive Method
Minimal detectable charge	≈3 pC	≈5–6 pC
Sensitivity	reference	+30–40% higher at testing voltage amplitude
Peak-to-mean ratio	8–9	7–13
Dominant charge range	5–10 pC	20–25 pC

## Data Availability

No new datasets were generated or analyzed during the current study. All data supporting the findings are included within the article.

## Abbreviations

The following abbreviations are used in this manuscript:

PD	Partial discharges
